# Active Learning in Veterinary Anatomy Education: Investigating the Impact of Peer-Led Q&A Games and Multimedia on Student Perceptions

**DOI:** 10.3390/vetsci12121174

**Published:** 2025-12-09

**Authors:** Alejandra Escudero, María Socorro Simó-Martínez, María José Morera, Ana Navarro-Serra, María García-Manzanares

**Affiliations:** 1 Department of Animal Production and Health, Public Veterinary Health and Food Science and Technology, School of Veterinary Medicine, Universidad Cardenal Herrera-CEU, CEU Universities, Alfara del Patriarca, 46115 Valencia, Spain; alejandra.escudero@uchceu.es; 2 Department of Animal Medicine and Surgery, School of Veterinary Medicine, Universidad Cardenal Herrera-CEU, CEU Universities, Alfara del Patriarca, 46115 Valencia, Spain; maria.simo@uchceu.es (M.S.S.-M.); maria.morera@uchceu.es (M.J.M.)

**Keywords:** veterinary, anatomy, multimedia, teaching, gamification

## Abstract

This study explores the pedagogical impact of a student-led teaching strategy in veterinary education, centered on the implementation of a monitored question-and-answer (Q&A) game integrated with multimedia content. Conducted in a classroom setting, the intervention encouraged students to actively formulate and pose questions to their peers—questions that could potentially appear on future assessments. The sessions were recorded and transformed into multimedia resources, enabling content review beyond the classroom. Results indicate a high student participation during practical activities (89.33%), as well as enhanced engagement with course material outside scheduled sessions. Students’ perceptions were collected in a survey (Cronbach’s alpha 0.920), revealing that more than sixty percent of answers fostered collaborative learning and anatomy assessments preparation. Moreover, fifty percent of students affirmed that the activity enhanced their participation and motivation and stimulated peer-to-peer interaction. These results could suggest that gamified student-led questioning combined with digital content creation can effectively support active learning and deeper understanding in veterinary anatomy education.

## Simple Summary

Veterinary anatomy is fundamental to training, yet its complexity and the volume of material often make it challenging for first-year students. These challenges often lead to gaps in understanding and reduced confidence, making it essential to explore strategies that promote active engagement and deeper learning. This study introduced an interactive approach where students created exam-style questions and multimedia resources as part of a question-and-answer tournament designed to make learning more dynamic and collaborative. The goal was to encourage participation, improve motivation, and support the development of skills such as teamwork and critical thinking. Findings suggest that the activity was well received, with students reporting increased motivation, better attendance, and perceived improvements in understanding. For instructors and curriculum designers, these results highlight the potential of game-based and peer-driven methods to complement traditional teaching, foster engagement, and strengthen essential competencies in veterinary education.

## 1. Introduction

The current educational environment is characterized by rapid and constant transformation, driven by technological advances and evolving modes of knowledge acquisition. In this context, traditional teaching methodologies are often perceived as ineffective, passive, and unmotivating, leading to reduced levels of student participation and engagement [1,2,3,4,5]. This scenario poses a significant challenge for educators who must adapt their pedagogical strategies and incorporate innovative approaches capable of fostering more effective learning, particularly in highly complex courses [6]. In this regard, technology-enhanced learning emerges as a promising alternative by offering more immersive and interactive learning experiences [7]. Specifically, game-based methodologies—focused on problem solving, teamwork, and communication—have proven more effective in engaging students and improving academic performance [6]. Likewise, blended learning approaches that combine face-to-face and online instruction have been shown to enhance time management, motivation, and personalized learning [8].

To foster active student participation, it is essential to combine traditional lectures with innovative and dynamic methodologies that promote critical and in-depth learning [9]. Such approaches not only encourage reflection and independent analysis but also reinforce collaboration and teamwork—key components of the educational process—leading to improved outcomes compared with traditional lecture-based instruction. A substantial body of research supports the view that active student engagement and collaborative learning are strongly associated with increased commitment and improved knowledge retention [10,11,12]. In this sense, the transition from a teacher-centered to a student-centered learning model has proven effective for developing the competencies and knowledge required in higher education [13,14,15]. This methodological shift entails establishing a self-directed learning environment in which students play a leading role in their own development, supported by continuous assessment techniques that promote ongoing feedback and individualized progress. Furthermore, involving students in designing and evaluating the learning process can significantly enhance motivation and academic performance [16,17].

The use of supplementary materials—such as images, videos, and interactive activities—further enriches the learning process for both students and instructors. Emerging technologies, including QR codes, enable the integration of dynamic content such as recorded Q&A sessions or links to additional resources, thereby expanding the capabilities of educational games and fostering deeper engagement [18,19].

Veterinary medicine is, among the academic programs, associated with the highest levels of stress, often accompanied by anxiety in these students [20]. For this reason, it is essential to provide a positive learning environment that helps reduce stress and supports their well-being while fostering effective knowledge acquisition [21]. Within the Veterinary Medicine curriculum, the development of transversal competencies is considered essential. One of the specific objectives of student training is to promote integration, communication, and application of knowledge in future clinical or professional contexts. Students must understand that formulating questions fosters reflection on the concepts studied, and that participating collaboratively in team-based tournaments helps consolidate knowledge and facilitate group integration [2,3,4,5].

The study presented here follows a cross-sectional design and can be applied to subjects within the Veterinary Medicine degree that incorporate workshops or seminars requiring active student participation, where course content is assessed through written or oral examinations [22,23,24,25,26].

Beyond improving academic performance, this project seeks to foster the comprehensive development of students by strengthening a set of transversal competencies that are essential in both academic and professional contexts [27,28,29,30]. The formulation of challenging, exam-type questions requires students to engage in processes of selection, organization, and synthesis of information, which stimulates higher-order cognitive skills and promotes a global and integrative understanding of the subject matter [31,32,33]. In parallel, the need to articulate questions clearly and coherently contributes to the refinement of communication skills, while the collaborative nature of the task reinforces students’ ability to negotiate, distribute responsibilities, and work collectively toward a shared intellectual product [34,35,36].

These pedagogical elements are closely aligned with workplace expectations in contemporary veterinary practice, where innovation, teamwork, problem-solving, and precise communication are increasingly valued competencies [34]. Moreover, the incorporation of multimedia components—particularly student-generated videos used for exam preparation—fosters creativity, digital literacy, and autonomous learning, enriching the educational experience and supporting the development of skills that extend beyond disciplinary knowledge [37,38,39,40].

The present work examines students’ perceptions of the anatomy designed game, with particular attention to how the activity influenced their engagement, teamwork dynamics, and preparation for assessments. Furthermore, the study explores whether generating and responding to questions helped students perceive the course more globally and consolidate concepts in a dynamic and practical manner. It also investigates the extent to which students found the activity enjoyable, whether it stimulated participation in practical sessions, and how they valued the videos as tools for exam preparation. In addition, students’ willingness to participate in similar activities in future courses provides insight into the sustainability and scalability of the approach.

This study aimed to evaluate the effectiveness of the Vet Academic Challenge as an active learning strategy within first-year veterinary anatomy courses. Specifically, the objectives were to assess students’ overall satisfaction with the activity and their perceived gains in engagement, motivation, teamwork, and conceptual understanding. Additionally, we intended to compare practical examination scores between two academic years (2023/2024, previous course, and 2024/2025, where this work was developed) to explore potential performance differences associated with the implementation of the activity. By addressing these goals, the study seeks to provide a comprehensive evaluation of students’ experiences and to determine the extent to which the Vet Academic Challenge supports both cognitive and non-cognitive competencies recognized as essential for veterinary education and practice.

## 2. Materials and Methods

### 2.1. Study Design

This study employed a cross-sectional observational design to evaluate veterinary students’ perceptions of a peer-led, game-based learning activity known as “Vet Academic Challenge”. The intervention was implemented during practical sessions in the first-year anatomy courses Structure and Function I at CEU Cardenal Herrera University (Valencia, Spain) during the complete 2024–2025 academic course.

**Figure 1:**
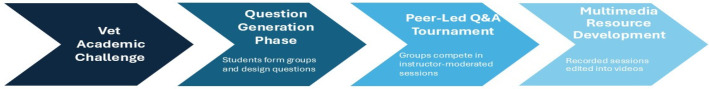
Main phases of the study.

The pedagogical strategy required students to work in small groups to create exam-type questions, present them to their peers in a competitive Q&A tournament, and subsequently generate multimedia resources based on the recorded sessions (Figure 1). Participation in the activity was voluntary and did not influence course grading.

The study population consisted of first-year veterinary students enrolled in the French-language group of the course Structure & Function I (*n* = 75). After completion of the activity, all students, both participants and non-participants, were invited to complete an anonymous online survey. It is important to note that not all students who took part in the project responded to the survey, and conversely, some students who did not participate in the project completed the questionnaire. Therefore, the variables of project participation and survey response do not fully overlap in size or composition.

### 2.2. Educational Intervention

The classes were conducted as part of the regular animal anatomy course. At the end of each class, in the last few minutes, the students were told to work in groups and create exam-like questions.

The Vet Academic Challenge consisted of three structured phases:

Question generation phase: Students were organized into groups of 4–6 and instructed to design challenging, syllabus-aligned questions requiring conceptual understanding, integration of anatomical knowledge, and higher-order reasoning. An example of a question is: “Identify the glenoid cavity in equine”. Anatomy professors supervised all the question generation process and assisted students in the creation of the videos.

Peer-led Q&A tournament: Groups competed by posing their questions to peers in a structured game format moderated by instructors. The sessions were recorded for pedagogical use.

Multimedia resource development: Recorded Q&A sessions were edited into short videos of one minute or less in duration and uploaded to the institutional SharePoint (Microsoft Corporation, Redmond, WA, USA) platform. Students could access these materials for exam preparation and review.

No external tools beyond institutional digital platforms like Blackboard Collaborate, (Anthology Inc., Boca Raton, FL, USA) or SharePoint were required for the study. Although artificial intelligence (AI)-assisted tools were permitted, as described in previous studies [41], they were not formally evaluated in this research.

### 2.3. Survey Instrument

Student perceptions of the educational intervention were assessed using an anonymous online questionnaire specifically designed for this study. The instrument consisted of demographic and previous experience questions and eight Likert-scale items labeled in this study as Q1–Q8 (1 = strongly disagree; 2 = disagree; 3 = neutral; 4 = agree; 5 = strongly agree) that aimed at evaluating key dimensions related to the Vet Academic Challenge, perceived understanding of anatomical concepts, usefulness of the activity for examination preparation, engagement during the practical sessions, motivation, enjoyment, and teamwork. The survey was available during September and October 2025. These items were developed to capture students’ subjective experiences and their appraisal of the pedagogical effectiveness of the activity (Supplementary Material S1). These were the questions in the survey:

1. Indicate your highest academic course.
2. Where are you from?
3. Have you ever participated in academic championships?
4. Have you ever recorded and edited academic videos?
5. Elaboration of questions helped me with the whole vision of the subject. (Q1)
6. Elaboration of exam-like questions motivated my participation. (Q2)
7. Reviewing videos helped me with my exam preparation. (Q3)
8. This challenge has improved my teamwork ability. (Q4)
9. This challenge allowed me to reinforce concepts in a practical and dynamic way. (Q5)
10. The time spent creating and answering questions was enjoyable. (Q6)
11. I would be happy to repeat this activity in other courses. (Q7)
12. Participating in this activity has been a positive incentive to attend practical sessions and workshops. (Q8)

In addition to the core perception items, the questionnaire included a binary question asking students whether they had participated in the Vet Academic Challenge (“yes”, “no”, or “maybe”). This allowed differentiation between respondents who were directly involved in the intervention and those who were not, enabling comparative analyses between groups. No identifying information was collected, ensuring full anonymity.

### 2.4. Data Collection

The survey was distributed online using Microsoft Forms after the completion of the academic course. Students were informed about the purpose of the study and the anonymous nature of their responses. Only fully completed forms were included in the analysis.

### 2.5. Statistical Analysis

All statistical analyses were performed using R software (version 4.3.1; R Core Team, 2023). Prior to analysis, the dataset was examined for completeness and consistency; responses coded as “0” were treated as missing values, as the students did not answer those questions.

Descriptive statistics were computed for all variables. For the eight Likert-scale items, results are presented as means, standard deviations, medians, and ranges, while categorical variables are summarized as frequencies and percentages.

The internal consistency of the perception questionnaire was assessed using Cronbach’s alpha, with values ≥ 0.70 considered indicative of acceptable reliability. Data normality was assessed with the Shapiro–Wilk test. Group comparisons between participating and non-participating students were conducted using independent-samples *t*-tests when normality assumptions were met, and Mann–Whitney U tests when distributions were non-normal. Statistical significance was set at *p* < 0.05 for all analyses.

To explore relationships between students’ perceptions, Pearson correlation coefficients were calculated in Likert-scale items.

### 2.6. Ethical Approval

The study was approved by the Ethics Committee of CEU Cardenal Herrera University (Reference: CEEI25/688, 25 July 2025). Participation was voluntary, and all procedures adhered to institutional and European ethical guidelines for research in education.

## 3. Results

### 3.1. Participant Profile

**Figure 2:**
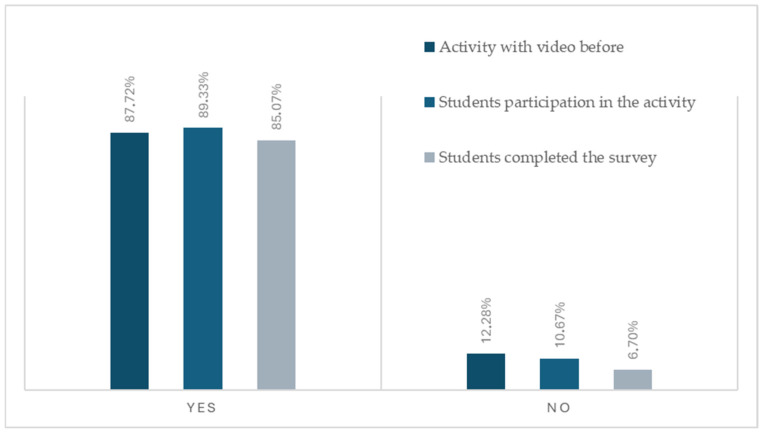
Participation and previous video experience of students.

All students enrolled in the French-language group of the course Structure and Function I (*n* = 75) were considered eligible for inclusion in the study, regardless of their participation in the educational project, as the perspectives of students who voluntarily chose not to engage in the activity were also deemed relevant. This inclusive approach enabled comparison between participants and non-participants and allowed examination of whether involvement in the project was associated with improved academic performance. Regarding participation, 89.33% of students reported having taken part in the activity and 10.67% indicated that they had not participated or did not remember, reflecting some uncertainty about their involvement (Figure 2). A total of 57 students completed the survey out of 67 that participated in the activity, yielding a response rate of 85.07%. 10 students did not answer the survey. To assess the reliability of the instrument, the internal consistency of the eight-item questionnaire was evaluated using Cronbach’s alpha. The questionnaire demonstrated excellent internal consistency, with a Cronbach’s alpha of 0.920 (95% CI = 0.876–0.947), indicating a high degree of coherence among items and supporting the suitability of the instrument for assessing students’ perceptions of the project. Previous experience in academic multimedia development was reported in 87.72% of students.

### 3.2. Motivation and Engagement: Questions 2 to 6

Motivation, engagement, and enjoyment in the learning process were analyzed through direct questions. Students were asked to indicate their level of agreement with several statements. Results in question 2, “Elaboration of exam-like questions motivated my participation,” showed that more than fifty percent of students agreed or strongly agreed with the statement (52.3%) and only one student did not answer to this question. Question 3, “Reviewing videos helped me with my exam preparation” received the highest number of “strongly agree” responses (38.7%). For question 4 “The challenge allowed me to improve my teamwork skills,”, question 5 “The challenge helped me consolidate concepts in a practical and dynamic way,” and question 6 “The time devoted to creating and solving questions was enjoyable,” more than 50% of the answers were distributed between “agree” and “strongly agree” (Table 1).

### 3.3. Collaboration and Future Willingness: Questions 7 and 8

The survey also included items assessing students’ willingness to engage in similar projects in future courses, as well as their perception of whether the activity improved their attention and participation during class sessions. Question 7, “I would participate in this activity again in other subjects”, assessed students’ intention to take part in similar activities in other subjects, to which 66.5% of respondents selected “neutral” or “agree”. In contrast, this question received the highest proportion of “disagree” and “strongly disagree” answers, with a total of 22.7%. Question 8, “Participation in this activity served as a positive stimulus for attending practical sessions and workshops”, focused on whether the activity positively influenced students’ attendance and engagement in practical sessions and workshops, with 68.4% of responses falling within the “neutral” or “agree” categories (Table 1).

### 3.4. Correlation Between Analyzed Items

The correlation analysis was carried out with all the questions of the survey and their corresponding answers. This revealed several strong and meaningful relationships among the perception items. Item Q8 (“willingness to participate in similar activities in the future”) emerged as the most central variable in the dataset, showing the highest correlations with multiple items, particularly Q7, Q4, Q5, and Q1. The strongest association was observed between Q7 and Q8 (r = 0.78), indicating that students who reported high enjoyment or motivation during the activity were also the most willing to engage in similar methodologies in other subjects.

Q8 also demonstrated substantial correlations with Q4 (r = 0.73), Q1 (r = 0.71), and Q5 (r = 0.67), suggesting that students’ willingness to repeat the activity was closely linked to their perceived learning, usefulness for exam preparation, and perceived contribution to understanding anatomical content. These findings highlight Q8 as a global indicator of the overall acceptance and perceived value of the Vet Academic Challenge.

Item Q7 also proved to be highly influential, exhibiting consistent correlations with several items. Beyond its strong association with Q8, Q7 correlated moderately to strongly with Q3 (r = 0.65), Q4 (r = 0.62), and Q5 (r = 0.63), reflecting a coherent pattern between enjoyment/motivation and perceived educational benefits, teamwork, and engagement (Table 2).

Additional moderate associations were found between Q3 and Q5 (r = 0.66), indicating that students who perceived teamwork positively also tended to view the activity as useful for exam preparation. Overall, the correlation matrix suggests a cohesive and interconnected structure in students’ perceptions, with motivation (Q7) and willingness to repeat the activity (Q8) acting as central nodes that integrate affective, cognitive, and collaborative dimensions of the learning.

### 3.5. Academic Assessment Results and Relationship with Questions 1 and 3

Anatomy practical exam grades were compared between academic courses 2023–2024 and 2024–2025. Results showed slightly increased mean grade in the last year (8.26 in 2023–2024 vs. 8.5 in 2024–2025), although no statistical differences were found (*p* = 0.37). The number of students enrolled in the subject was higher during the 2024–2025 academic year (*n* = 75 vs. *n* = 67 in 2023–2024); therefore, we can hypothesize that more variability could be present in the group. Statements in questions 1 and 3 (“Elaboration of questions helps me with the whole vision of the subject” and “Reviewing videos helped me with my exam preparation”, respectively) showed that 60% of students agreed or strongly agreed with these affirmations (Table 1).

## 4. Discussion

### 4.1. Overall Perceptions of Educational Intervention

The present study aimed to evaluate veterinary students’ perceptions of a peer-led, game-based learning strategy through the Vet Academic Challenge. Overall, the results indicate that students reported positive perceptions regarding their understanding of course content, engagement, teamwork, and motivation. These findings align with previous studies that highlight the value of active learning strategies in veterinary and medical education.

Although several authors argue that student-centered approaches foster deeper conceptual understanding and metacognitive engagement, our study did not directly examine higher-order thinking or metacognitive processes [42]. Nevertheless, the improvement in perceived understanding reported by participants aligns with existing evidence, suggesting that activities involving problem formulation and collaborative reasoning may indirectly activate such cognitive mechanisms and contribute to more meaningful anatomical learning.

The overall positive response to the intervention also mirrors findings from studies incorporating gamification, role-play, and alternative methodologies in veterinary curricula. For instance, students often perceive active methodologies as more stimulating and more relevant to clinical reasoning development [43]. This alignment supports the integration of interactive learning practices as part of preclinical anatomy training, emphasizing that the perceived value of the activity is not isolated but reflects a wider trend in contemporary veterinary pedagogy [44].

### 4.2. Perception of Question Generation and Cognitive Engagement

One of the core components of the Vet Academic Challenge involves having students create exam-type questions. Our data show that students valued this process as a meaningful contribution to their learning. These findings support the notion that question generation enhances metacognitive awareness and mastery of concepts, as demonstrated in educational frameworks emphasizing student-centered approaches [45]. Creating questions requires analytical processing and encourages learners to identify key concepts, construct meaningful associations, and anticipate common misconceptions.

Comparable results are found in methodologies involving student-authored assessment tasks, role-based gamification, or case-based learning, all of which have shown positive effects on knowledge integration and critical thinking in health sciences education [43]. Additionally, peer-led questioning resonates strongly with the principles underpinning problem-based learning, where active participation and inquiry stimulate deeper learning [42].

### 4.3. Motivation, Enjoyment, and Student Engagement

Students expressed strong agreement regarding their motivation and enjoyment during the activity. The literature supports the idea that gamified environments and competitive elements enhance attention, commitment, and emotional engagement. For example, studies on interactive veterinary learning and collaborative environments have shown that students generally appreciate dynamic formats that deviate from traditional lectures and enhance motivation [46].

The correlation analysis in our study further reflects this trend, with positive associations between motivation, enjoyment, willingness to repeat the activity, and perceived learning. Similar relationships have been documented in studies investigating ICT-supported learning experiences in veterinary medicine, where increased enjoyment predicts higher engagement and greater perceived value [47].

A particularly relevant finding of this study is the strong and coherent pattern observed in the correlation matrix, where Q8 and Q7 act as central determinants integrating the cognitive, affective, and collaborative dimensions of the learning experience. The very high correlation between enjoyment/motivation (Q8) and willingness to participate in similar activities (Q7) (r = 0.78) suggests that students’ emotional engagement is a key predictor of future acceptance of innovative methodologies. Moreover, the substantial associations between Q8 and teamwork (Q4; r = 0.73), understanding of anatomical concepts (Q1; r = 0.71), and perceived usefulness for exam preparation (Q5; r = 0.67) reinforce the idea that students who recognize academic value in the activity are more likely to endorse its repetition. Likewise, the consistent correlations of Q7 with video usefulness (Q3; r = 0.65), usefulness of the activity (Q5; r = 0.63), and teamwork (Q4; r = 0.62) indicate that motivation is deeply intertwined with collaborative and cognitive benefits. Together, these results underscore that both motivational and acceptance-related factors are central drivers of students’ overall experience with the Vet Academic Challenge, echoing prior findings that engagement, perceived utility, and emotional involvement are core determinants of success in active learning environments.

### 4.4. Teamwork and Peer Collaboration

Students also indicated that teamwork played an essential role in the learning process. The high correlations found in our dataset between teamwork, willingness to repeat the activity, and understanding of content echo findings in studies highlighting the benefits of collaborative learning environments. In veterinary anatomy and other preclinical subjects, collaborative problem-solving has been shown to reinforce communication skills, encourage critical thinking, and improve students’ sense of responsibility and autonomy [9].

Moreover, the structured nature of the Vet Academic Challenge, with defined roles and group responsibilities, reflects the broader educational movement encouraging peer-supported and inquiry-based tasks as part of veterinary education reform [1].

### 4.5. Usefulness of Multimedia Resources

A key contribution related to this project lies in the creation of digital video resources derived from the recorded Q&A sessions. The students’ responses showed positive perceptions regarding the usefulness of these videos for exam preparation. This aligns with the increased adoption of multimedia in veterinary education, particularly as a strategy to supplement hands-on anatomical training [1,48].

Furthermore, research on interactive videos shows improved acceptance and enhanced conceptual understanding when students can revisit class material asynchronously [49]. The perceived usefulness of videos in supporting practical learning reported by students appears to align with patterns described in flipped classroom models, where multimedia resources can enhance preparation and comprehension. In our study, the item that received the highest proportion of strongly agree responses, “Reviewing the videos helped me with my exam preparation”, could indicate that students highly valued the creation of multimedia content as a resource they can revisit at home to consolidate their learning. However, as our study did not directly analyze usage data, the relationship with multimedia benefits in education and the average number of views per video should be interpreted cautiously and considered as indicative rather than conclusive.

### 4.6. Willingness to Participate in Future Activities

Most students reported that they would like to participate in similar activities in other subjects. This finding is consistent with literature showing high student willingness to engage in innovative teaching practices when they perceive them as relevant, clear, and beneficial [50]. Despite the overall positive results, a small group of students responded negatively to the disposition to participate in future activities in a higher proportion than for the other questions. This could be due to differences in individual learning preferences, varying levels of confidence or familiarity with multimedia creation, or perceptions of the activity as overly time-consuming compared with more traditional study methods.

The general acceptance suggests that the Vet Academic Challenge may serve as a scalable model for integrating active methodologies more broadly into veterinary curricula. The activity’s emphasis on peer instruction, competition, and multimedia resources aligns with current trends in educational innovation within veterinary programs [51].

### 4.7. Limitations

This study presents several limitations that should be considered when interpreting the findings. Outcomes were based on self-reported perceptions, which may introduce response bias and limit the ability to establish causal relationships. Incorporating objective endpoints, such as pre/post knowledge assessments or practical exam performance, would strengthen future analyses. The research was conducted within a single course at one institution, which may restrict the generalizability of the results to other contexts. Voluntary participation could also have introduced selection bias toward more motivated students, and the absence of a parallel control group receiving exclusively traditional instruction constrains comparative interpretation.

Although the questionnaire demonstrated high internal consistency, it has not yet undergone full psychometric validation, which may affect the robustness and applicability of the instrument. Future studies should include formal validation procedures to ensure accurate measurement of the intended constructs across different cohorts and settings. Additionally, while students reported that videos were highly useful, engagement analytics such as watch time were not quantified; only an approximate average of 50 views per video was noted. This limits the ability to explore dose–response effects or confirm whether perceived usefulness reflects actual utilization.

Finally, although the activity was designed to promote collaborative reasoning and problem formulation—processes associated with higher-order thinking and metacognitive engagement—these cognitive dimensions were not directly measured. Further research should incorporate validated tools to assess metacognition and critical thinking, as well as additional variables such as stress and anxiety, to provide a more comprehensive understanding of the intervention’s impact [10].

### 4.8. Future Research Directions

Future research should expand on these findings by incorporating larger and more diverse student cohorts, including multiple language groups, institutions, and academic years, to improve the external validity of the results. Longitudinal designs would be particularly valuable to assess whether improvements in perceived understanding, motivation, and teamwork translate into sustained learning gains and enhanced academic performance over time.

Further studies should also integrate objective learning outcomes, such as exam scores, performance in practical assessments, or concept retention tests, to determine the actual cognitive impact of student-generated questions and game-based learning. Experimental or quasi-experimental designs comparing the Vet Academic Challenge with traditional instruction or other active-learning strategies would help clarify its specific benefits.

Additionally, qualitative approaches—such as focus groups or semi-structured interviews—could yield deeper insights into the mechanisms by which students perceive the activity as helpful (e.g., improved autonomy, increased confidence, peer accountability). Since the current study suggests a strong perceived value in multimedia resources, future research should also examine which types of video or digital materials (short clips, annotated recordings, spaced microlearning formats) most effectively enhance learning.

Finally, as artificial intelligence tools increasingly permeate educational environments, studies could explore how AI-assisted question creation, feedback generation, or automated content analysis may complement or enhance student-driven activities such as the Vet Academic Challenge.

## 5. Conclusions

The Vet Academic Challenge was positively received by first-year veterinary students, who reported perceived gains in engagement, motivation, teamwork, and understanding of anatomical content. Questionnaire consistency and correlations suggest that the activity may have supported a coherent learning experience and students valued the multimedia resources produced. While these findings are based on self-reported perceptions and a limited sample, they align with literature on active, collaborative learning. It is important to note that no significant differences were observed in practical exam scores between academic years with and without activity. This underscores the need for more objective measures to evaluate the cognitive impact of the intervention, as exam performance alone may not capture nuanced learning outcomes. Future studies will incorporate controlled comparisons and explore additional variables (including stress and anxiety) that may influence students’ learning experience. Overall, the Vet Academic Challenge appears to be a feasible and adaptable strategy within veterinary curricula.

## Figures and Tables

**Table 1:** Student feedback—percentage distribution.

Question	1 (Strongly Disagree)	2 (Disagree)	3 (Neutral)	4 (Agree)	5 (Strongly Agree)
Q1	3.4%	6.9%	25.8%	44.8%	15.5%
Q2	3.2%	9.6%	25.8%	27.4%	24.9%
Q3	8.0%	8.0%	17.7%	27.7%	38.7%
Q4	0.0%	19.3%	35.0%	36.8%	8.7%
Q5	0.0%	7.0%	35.0%	36.8%	21.0%
Q6	3.5%	14.0%	28.0%	38.6%	15.7%
Q7	5.2%	17.5%	31.5%	35.0%	10.5%
Q8	5.2%	15.7%	29.8%	38.6%	10.5%

**Table 2:** Descriptive statistics and correlations.

Questions	Correlation (r)								Mean (M)	SD
	Q1	Q2	Q3	Q4	Q5	Q6	Q7	Q8		
Q1	1.00	0.53	0.41	0.53	0.55	0.54	0.55	0.70	3.58	1.07
Q2	0.53	1.00	0.34	0.53	0.43	0.52	0.63	0.57	3.60	1.19
Q3	0.41	0.34	1.00	0.41	0.66	0.32	0.65	0.53	3.88	1.23
Q4	0.53	0.53	0.41	1.00	0.64	0.63	0.61	0.73	3.35	0.90
Q5	0.55	0.43	0.66	0.64	1.00	0.54	0.62	0.66	3.72	0.88
Q6	0.54	0.52	0.32	0.63	0.54	1.00	0.57	0.64	3.49	1.04
Q7	0.55	0.63	0.65	0.61	0.62	0.57	1.00	0.78	3.28	1.05
Q8	0.70	0.57	0.53	0.73	0.66	0.64	0.78	1.00	3.33	1.04

Note. All correlations are significant at *p* < 0.01.

## Data Availability

The data presented in this study are available upon request from the corresponding authors (M.G.M. and A.N.S.). The reason for this restriction is that the data are confidential.

## Supplementary Materials

The following supporting information can be downloaded at: https://www.mdpi.com/article/10.3390/vetsci12121174/s1, Supplementary Material S1: Survey.

## Abbreviations

The following abbreviations are used in this manuscript:

AI	Artificial Intelligence
CBVE	Competency-Based Veterinary Education
CEU	Universidad Cardenal Herrera—CEU Universities
OECD	Organisation for Economic Co-operation and Development
Q&A	Question and Answer
Q1–Q8	Questionnaire Items 1 to 8
R	R Statistical Software
