# Supplementary material for: Active Learning in Veterinary Anatomy Education: Investigating the Impact of Peer-Led Q&A Games and Multimedia on Student Perceptions
Source: Vet Sci. 2025 Dec 9;12(12):1174. doi: 10.3390/vetsci12121174 (PMC12737326; doi:10.3390/vetsci12121174)
Supplement: Supplementary file 1 [file vetsci-12-01174-s001.zip › vetsci-4001434-supplementary.pdf]

Encuesta participantes ***Vet Academic Challenge***

Os invitamos a participar en la encuesta sobre el proyecto que se ha desarrollado en las prácticas y talleres, donde nos hemos centrado en la labor de **alumnos como profesores en** temas como **elaboración de preguntas de examen**, creación y edición de material docente en **vídeo** y juegos en **equipo** para evaluar conocimientos. Esta encuesta es voluntaria y anónima. ------ Nous vous invitons à participer à l'enquête sur le projet qui a été développé lors des travaux pratiques et des ateliers, où nous nous sommes concentrés sur le rôle des **étudiants en tant qu'enseignants** dans des domaines tels que la **rédaction de questions d'examen,** la création et l'édition de matériel pédagogique en **vidéo,** ainsi que les jeux en équipe pour évaluer les connaissances. -------- Cette enquête est volontaire et anonyme. We invite you to participate in the project survey about the **role of students as professors** in areas such as **exam question** writing, creation and editing of educational **video** materials, and **team-based games** for knowledge assessment. This survey is voluntary and anonymous

1

Indica el curso académico más alto en el que estás matriculado: Indique le niveau d'études le plus élevé dans lequel vous êtes inscrit(e):
Indicate your highest academic course:

2

¿De dónde eres? D'où venez-vous ?
Where are you from?

- ◯ Spain
- ◯ France
- ◯ Other

3

¿Has participado antes en torneos de preguntas o challenge académicos? Avez-vous déjà participé à des tournois de questions ou à des défis académiques ?
Have you ever participated in academic championships?

- ◯ Yes
- ◯ No
- ◯ Maybe

4

¿Has realizado vídeos anteriormente dentro del entorno académico?Avez-vous déjà réalisé des vidéos dans un cadre académique ? Have you ever recorded and edited academic videos?

- ◯ Yes
- ◯ No
- ◯ Maybe

5

Elaborar preguntas me permite ver la asignatura de forma global. Élaborer des questions me permet d'avoir une vision globale de la matière. Elaboration of questions helps me with the whole vision of the subject.

	Muy de acuerdo /Strongly agree	De acuerdo /Agree	Neutral	En desacuerdo /Disagree	Muy ene desacuerdo /Very disagree
Elige / Choose	○	○	○	○	○

6

Elaborar preguntas tipo examen me ha motivado a participar en esta actividad. Le fait d'élaborer des questions de type examen m'a motivé(e) à participer à cette activité.
Elaboration of exam-like questions motivated my participation.

	Muy de acuerdo /Strongly agree	De acuerdo /Agree	Neutral	En desacuerdo /Disagree	Muy en desacuerdo /Strongly disagree
Elige / Choose	○	○	○	○	○

7

Revisar los vídeos me ha ayudado a preparar el examen.
Le visionnage des vidéos m'a aidé à préparer l'examen.
Reviewing videos helped me with my exam preparation.

	Muy de acuerdo /Strongly agree	De acuerdo /Agree	Neutral	En desacuerdo /Disagree	Muy en desacuerdo /Strongly Disagree
Elige / Choose	◯	◯	◯	◯	◯

8

El challenge me permitió mejorar mi capacidad de trabajo en equipo. Le challenge m'a permis d'améliorer ma capacité à travailler en équipe. This challenge has improved my team-work hability.

	Muy de acuerdo /Strongly agree	De acuerdo /Agree	Neutral	En desacuerdo /Disagree	Muy en desacuerdo /Strongly disagree
Elige / Choose	◯	◯	◯	◯	◯

9

El challenge me permitió afianzar conceptos de manera práctica y dinámica. Le challenge m'a permis de consolider des concepts de manière pratique et dynamique.This challenge allowed me to reinforce concepts in a practical and dynamic way

	Muy de acuerdo /Strongly agree	De acuerdo /Agree	Neutral	En desacuerdo /Disagree	Muy en desacuerdo /Strongly disagree
Elige/Choose	◯	◯	◯	◯	◯

10

El tiempo dedicado a la creación y resolución de preguntas fue entretenido. Le temps consacré à la création et à la résolution de questions a été divertissant. The time spent creating and answering questions was enjoyable.

	Muy de acuerdo/ Strongly agree	De acuerdo/Agree	Neutral	En desacuerdo/Disag ree	Muy en desacuerdo/Stron gly disagree
Elige/Choose	◯	◯	◯	◯	◯

11

Volvería a hacer esta actividad en otras asignaturas. Je referais cette activité dans d'autres matières. I would be happy to repeat this activity in other courses

	Muy de acuerdo / Strongly agree	De acuerdo /Agree	Neutral	En desacuerdo /Disagree	Muy en desacuerdo /Strongly disagree
Elige / Choose	◯	◯	◯	◯	◯

12

La participación en esta actividad ha supuesto un estímulo positivo para asistir a las prácticas y talleres. La participation à cette activité a été une source de motivation positive pour assister aux travaux pratiques et aux ateliers. Participating in this activity has been a positive incentive to attend practical sessions and workshops.

	Muy de acuerdo/ Strongly agree	De acuerdo / Agree	Neutral	En desacuerdo / Disagree	Muy en desacuerdo / Strongly disagree
Elige / Choose	◯	◯	◯	◯	◯

13

Indica lo que quieras comentar acerca del challenge: **Indiquez vos impressions sur cette expérience:**
Indicate your thoughts about this experience:

Este contenido no está creado ni respaldado por Microsoft. Los datos que envíe se enviarán al propietario del formulario.

Microsoft Forms
